# Discrimination between leucine-rich glioma-inactivated 1 antibody encephalitis and gamma-aminobutyric acid B receptor antibody encephalitis based on ResNet18

**DOI:** 10.1186/s42492-023-00144-5

**Published:** 2023-08-18

**Authors:** Jian Pan, Ruijuan Lv, Qun Wang, Xiaobin Zhao, Jiangang Liu, Lin Ai

**Affiliations:** 1 School of Computer and Information Technology, Beijing Jiaotong University, Beijing, 100044 China; 2 Department of Neurology, Beijing Tiantan Hospital, Capital Medical University, China National Clinical Research Center for Neurological Diseases, Beijing, 100070 China; 3 Department of Nuclear Medicine, Beijing Tiantan Hospital, Capital Medical University, Beijing, 100070 China; 4 School of Engineering Medicine, Beihang University, Beijing, 100191 China; 5 Key Laboratory of Big Data-Based Precision Medicine (Beihang University), Ministry of Industry and Information Technology of the People’s Republic of China, Beijing, 100191 China

**Keywords:** ResNet18, Fluorodeoxyglucose-positron emission tomography, GABAB receptor antibody encephalitis, Deep learning, LGI1 antibody encephalitis

## Abstract

This study aims to discriminate between leucine-rich glioma-inactivated 1 (LGI1) antibody encephalitis and gamma-aminobutyric acid B (GABAB) receptor antibody encephalitis using a convolutional neural network (CNN) model. A total of 81 patients were recruited for this study. ResNet18, VGG16, and ResNet50 were trained and tested separately using 3828 positron emission tomography image slices that contained the medial temporal lobe (MTL) or basal ganglia (BG). Leave-one-out cross-validation at the patient level was used to evaluate the CNN models. The receiver operating characteristic (ROC) curve and the area under the ROC curve (AUC) were generated to evaluate the CNN models. Based on the prediction results at slice level, a decision strategy was employed to evaluate the CNN models’ performance at patient level. The ResNet18 model achieved the best performance at the slice (AUC = 0.86, accuracy = 80.28%) and patient levels (AUC = 0.98, accuracy = 96.30%). Specifically, at the slice level, 73.28% (1445/1972) of image slices with GABAB receptor antibody encephalitis and 87.72% (1628/1856) of image slices with LGI1 antibody encephalitis were accurately detected. At the patient level, 94.12% (16/17) of patients with GABAB receptor antibody encephalitis and 96.88% (62/64) of patients with LGI1 antibody encephalitis were accurately detected. Heatmaps of the image slices extracted using gradient-weighted class activation mapping indicated that the model focused on the MTL and BG for classification. In general, the ResNet18 model is a potential approach for discriminating between LGI1 and GABAB receptor antibody encephalitis. Metabolism in the MTL and BG is important for discriminating between these two encephalitis subtypes.

## Introduction

Autoimmune encephalitis (AE) is an immune-mediated disease in which antibodies act against neuronal synapses and cell surfaces [1, 2]. Autoimmune limbic encephalitis (ALE) is a common type of AE. A dramatic reduction in neuropsychiatric functions is a hallmark of ALE [3]. ALE has many subtypes, and leucine-rich glioma-inactivated 1 (LGI1) antibody encephalitis and gamma-aminobutyric acid B (GABAB) receptor antibody encephalitis are two typical subtypes [4]. Approximately 10% of LGI1 antibody encephalitis cases are associated with various cancers, such as thymoma [3, 5], and approximately half of GABAB receptor antibody encephalitis cases are associated with small-cell lung cancer [5], which is a common cause of death from cancer [6, 7]. Thus, early and accurate discrimination between LGI1 and GABAB receptor antibody encephalitis can inform different cancer screenings, thereby facilitating individualized treatment decisions and improving clinical outcomes. The diagnosis of LGI1 and GABAB receptor antibody encephalitis depends on antibody testing. However, antibody testing has two main shortcomings: it is time consuming and not easily accessible [5], which likely delays treatment. Previous studies have shown that early diagnosis and treatment can improve the clinical outcomes of patients with AE [8, 9]. A position study [5] stated that while waiting for the results of antibody testing, patients can initially be evaluated using commonly used diagnostic methods, such as magnetic resonance imaging (MRI), for preliminary treatment [5]. Furthermore, the sensitivity of positron emission tomography (PET) is higher than that of MRI for detecting LGI1 [10, 11] and GABAB receptor antibody encephalitis [12]. Therefore, PET is a potential imaging technique for differentiating these two types of encephalitis. However, the abnormal metabolisms of LGI1 and GABAB receptor antibody encephalitis in the PET images were similar. Previous studies found that the metabolism of the medial temporal lobe (MTL) and basal ganglia (BG) was abnormal in patients with LGI1 antibody encephalitis [13–15] and those with GABAB receptor antibody encephalitis [16–18]. Thus, it is difficult to discriminate between LGI1 and GABAB receptor antibody encephalitis based on visual interpretation of PET images. In clinical practice, visual interpretation is the traditional method of diagnosis using medical images [19]. However, this method depends on the clinician’s experience, which is subjective and inconsistent among clinicians [19]. Some subtle abnormal metabolisms of patients with AE in PET images can be ignored [20]. Fortunately, machine learning (ML) has been increasingly employed to analyze medical images and improve diagnoses [21]. Thus, ML is a potential method for discriminating between LGI1 and GABAB receptor antibody encephalitis based on PET images.

As a recently developed ML methodology, deep learning (DL) has been extensively used in medical image analyses, including classification [22], segmentation [23], and image registration [24]. In particular, it exhibited excellent performance in the intelligent analysis of PET images of patients with brain diseases. For example, Ding et al. [25] applied a convolutional neural network (CNN) model based on PET images to improve the detection of Alzheimer’s disease. Shen et al. [26] employed a modified group lasso sparse deep belief network model to discriminate patients with Parkinson’s disease from healthy participants based on PET images. These studies suggest that DL methods based on PET images can aid in the precise diagnosis of brain diseases. However, it remains unclear whether LGI1 and GABAB receptor antibody encephalitis can be accurately discriminated using DL models based on PET images.

This study aimed to construct CNN models with different convolutional layers based on PET images to discriminate between LGI1 and GABAB receptor antibody encephalitis. When the PET image passes forward through the convolutional layers, the extracted image features are refined to reflect subtle changes in the PET image. Thus, given the strong ability of CNN models to mine crucial features and subtle information, these models may accurately characterize the properties of PET images of both LGI1 and GABAB receptor antibody encephalitis. It was hypothesized that these CNN models could detect abnormal metabolism in PET images of these two types of encephalitis and thereby accurately discriminate them from each other.

## Methods

### Participants

The Medical Ethics Committee of Beijing Tiantan Hospital approved this study, which complied with the Declaration of Helsinki. The study recruited 81 patients, including 56 males and 25 females (mean ± SD age = 56.99 ± 12.70 years). Table 1 summarizes the patient demographics. The 81 patients fulfilled the following inclusion criteria: (1) antibody testing of cerebrospinal fluid (CSF) and/or serum was positive for LGI1 antibodies or GABAB receptor antibodies; (2) patients presenting clinical symptoms, such as memory impairments, seizures, and cognitive dysfunction; (3) PET and CT images were available.

**Table 1:** Clinical characteristics of patients

	LGI1 antibody encephalitis	GABAB receptor antibody encephalitis	*p* value
Age (year), mean ± SD	58.27 ± 12.62 (*n* = 64)	52.18 ± 12.19 (*n* = 17)	0.08^a^
Gender (male/female)	44/20	12/5	0.88^b^
Weight (kg), mean ± SD	70.28 ± 11.90 (*n* = 63)^c^	70.65 ± 10.06 (*n* = 17)	0.91^a^
Height (cm), mean ± SD	168.56 ± 7.15 (*n* = 63)^c^	169.06 ± 7.35 (*n* = 17)	0.80^a^

^a^Two-sample student test

^b^Pearson’s *χ*^2^ test

^c^The weight and height of one patient with LGI1 antibody encephalitis were unknown

Patients with seizures resulting from brain structural lesions (such as traumatic lesions, tumors, and stroke) or other diseases (such as severe hypo/hyperglycemia, malignant hypertension, or renal/hepatic failure) were excluded.

### Antibody testing

In this study, all the patients underwent antibody testing. The LGI1 antibody and GABAB receptor antibody in the CSF or serum were tested using cell-based assays and immunohistochemistry performed in the Laboratory of Neurological Immunology of Peking Union Medical College Hospital.

### Image acquisition and preprocessing

To acquire PET and CT images, all patients underwent a ^18^F-FDG PET/CT scan. Firstly, each patient was required to fast for at least six hours. Secondly, ^18^F-FDG (0.10–0.15 mCi/kg) was intravenously injected after a normal blood glucose level was confirmed. Thirdly, the patients rested for 30 min with their eyes open in a dark room. Brain PET/CT images were obtained using a GE Discovery 690 scanner. The acquisition parameters for the PET images were as follows: matrix = 192 $\times$ 192 pixels, voxel size = 1.5625 $\times$ 1.5625 $\times$ 3.2700 mm^3^, axial slides = 47.

Statistical parametric mapping software (SPM12; Wellcome Trust Center for Neuroimaging, London, UK) was used to preprocess all images. Firstly, for each patient, co-registration was performed between the CT and PET images. Secondly, a spatial normalization was performed from the co-registered CT images to the Montreal Neurological Institute (MNI) template using the Clinical Toolbox, which is an extension of SPM12 (https://www.nitrc.org/projects/clinicaltbx/) [27]. Thirdly, the spatial transformation of CT normalization was applied to PET, by which the PET images were adjusted to match the MNI template; then, the resolution of the PET images was changed to 2 × 2 × 2 mm^3^. Fourthly, a 6-mm isotropic full-width-half-maximum was used to smooth the normalized PET images. Fifthly, for each PET image, the mean value of the intensities across the highest 20% of voxels of the corresponding PET image [28] was used to normalize the smoothed PET image, by which the intensity of each voxel of the PET image was divided. Sixthly, the gray matter of the PET image was retained and used to extract image slices.

### Image slices extraction and augmentation

**Fig. 1:**
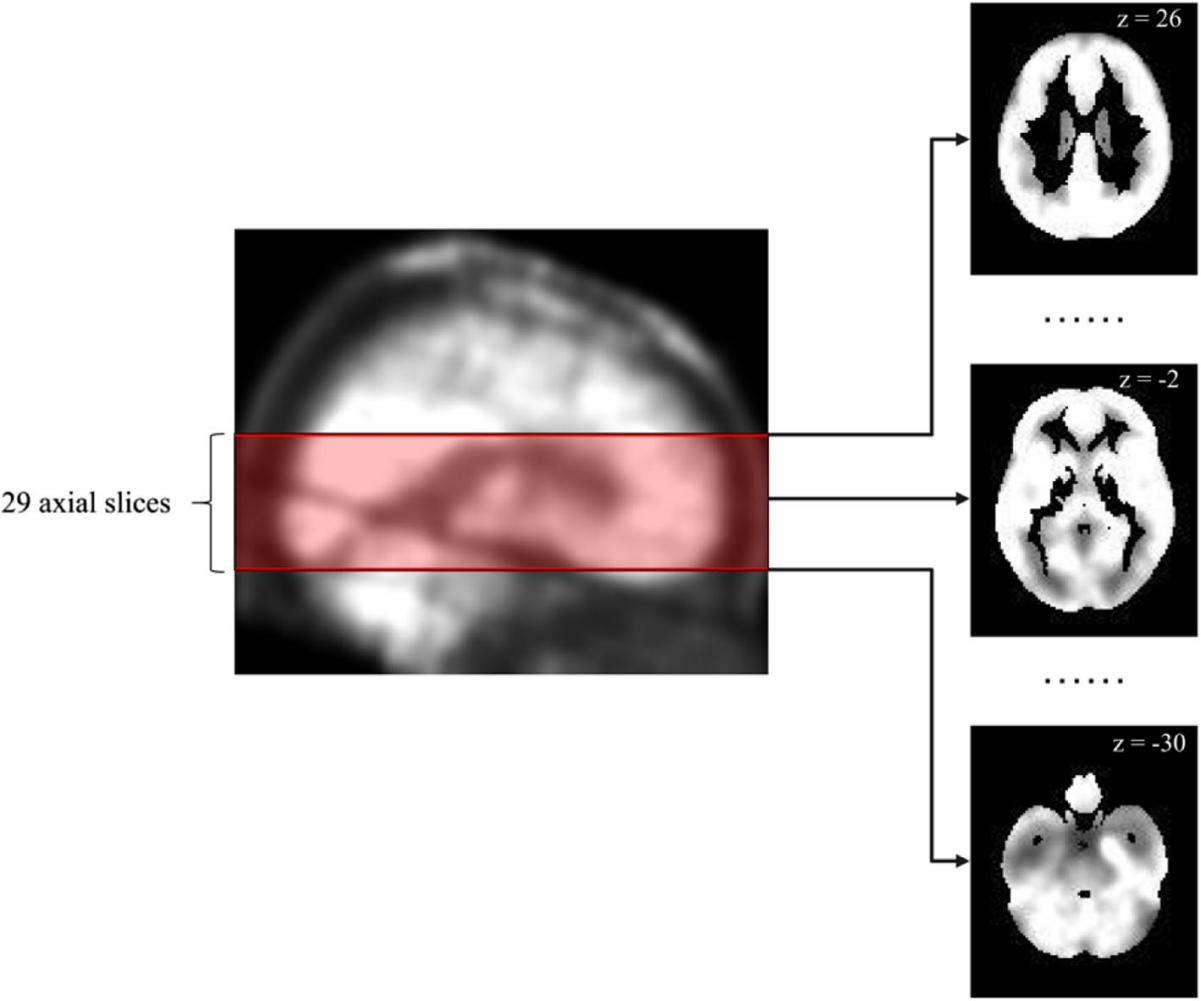
An example of axial image slices extraction. For each patient, 29 axial image slices were extracted from each preprocessed PET image. The left image was a brain PET image in sagittal view. The right images were the examples of the extracted axial image slices. The Z values in the right images indicate the MNI coordinates of axial image slices

**Fig. 2:**
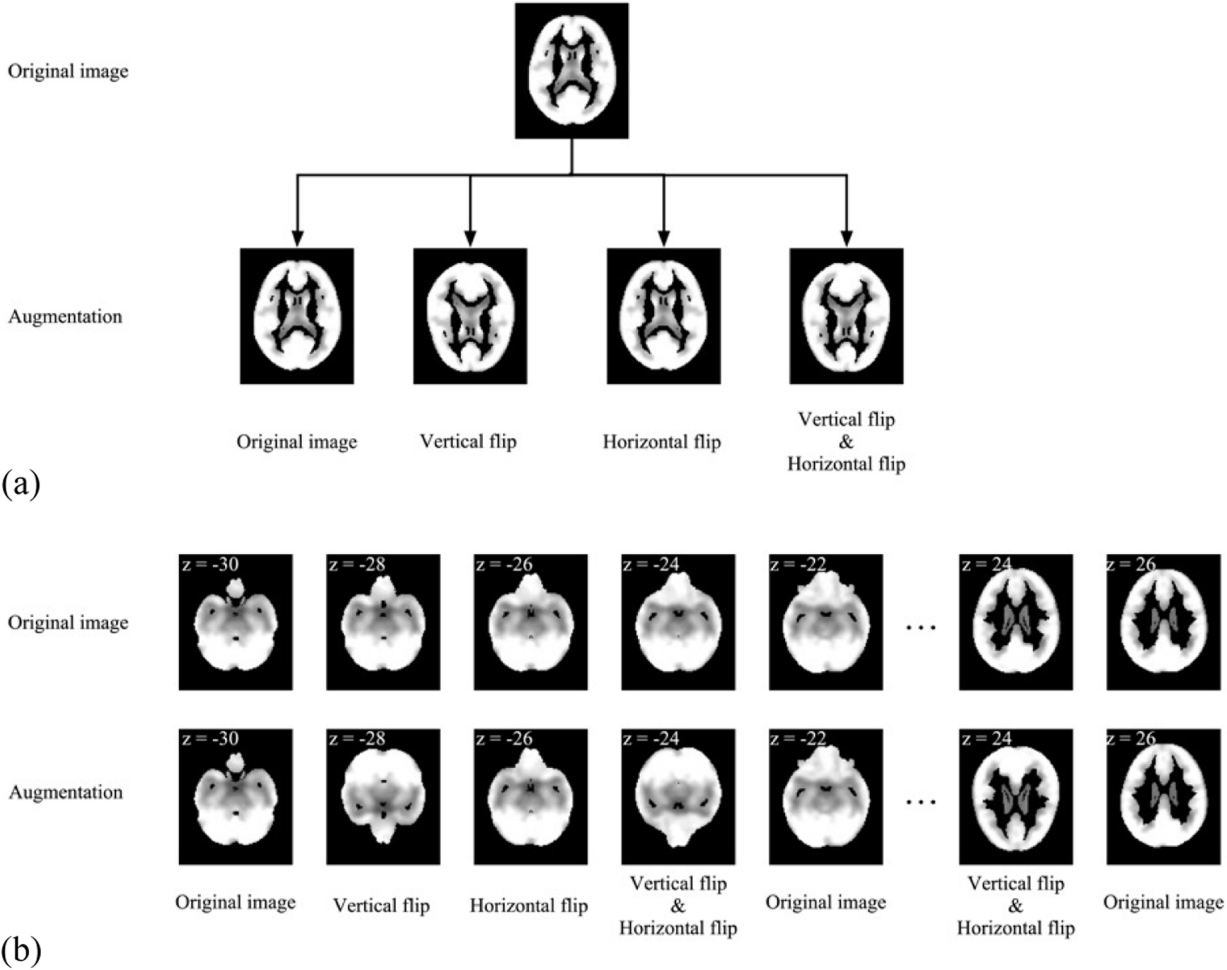
Illustrations of image slices augmentation. **a** Image augmentation for the GABAB receptor antibody encephalitis. Four types of augmentation were performed on each original image slice, namely, maintaining the original image, vertical flip, horizontal flip, and a combination of vertical and horizontal flips; **b** Image augmentation for the LGI1 antibody encephalitis. One of four types of augmentation was performed on the original image slices from bottom to top sequentially, namely, maintaining the original image, vertical flip, horizontal flip, and a combination of vertical and horizontal flips. For (**a**) and (**b**), the images in top row were original image slices and the images in bottom row were augmented image slices. The Z values in the image slices indicate the MNI coordinates of axial image slices

Multiple studies have shown that the MTL and BG metabolism is abnormal in patients with LGI1 antibody encephalitis [13–15] and those with GABAB receptor antibody encephalitis [16–18]. Based on the Human Brainnetome Atlas [29], there are 30 axial image slices containing the MTL or BG. One of these axial image slices was excluded because in this slice, only one voxel belonged to the MTL and no voxels belonged to the BG. Thus, as Fig. 1 shows, 29 axial image slices were extracted from the preprocessed PET image of each patient. Because the number of patients with GABAB receptor antibody encephalitis was relatively small, vertical and horizontal flips were used to augment the original image slices of all patients with GABAB receptor antibody encephalitis. As Fig. 2(a) shows, for GABAB receptor antibody encephalitis, four types of augmentation were performed for each original image slice: maintaining the original image, vertical flip, horizontal flip, and a combination of vertical and horizontal flips. Thus, the number of image slices with GABAB receptor antibody encephalitis was increased to four times. To maintain the consistency of the augmentation operation between LGI1 and GABAB receptor antibody encephalitis, four types of augmentation were applied to the original image slices of all patients with LGI1 antibody encephalitis. As Fig. 2(b) shows, for LGI1 antibody encephalitis, the image slices from bottom to top sequentially underwent one of four types of augmentation: maintaining the original image, vertical flip, horizontal flip, and a combination of vertical and horizontal flips. The augmented image slices were used to replace the original image slices. Therefore, the number of image slices with LGI1 antibody encephalitis remained unchanged. After augmentation, 3828 image slices were produced.

### CNN classification

**Fig. 3:**
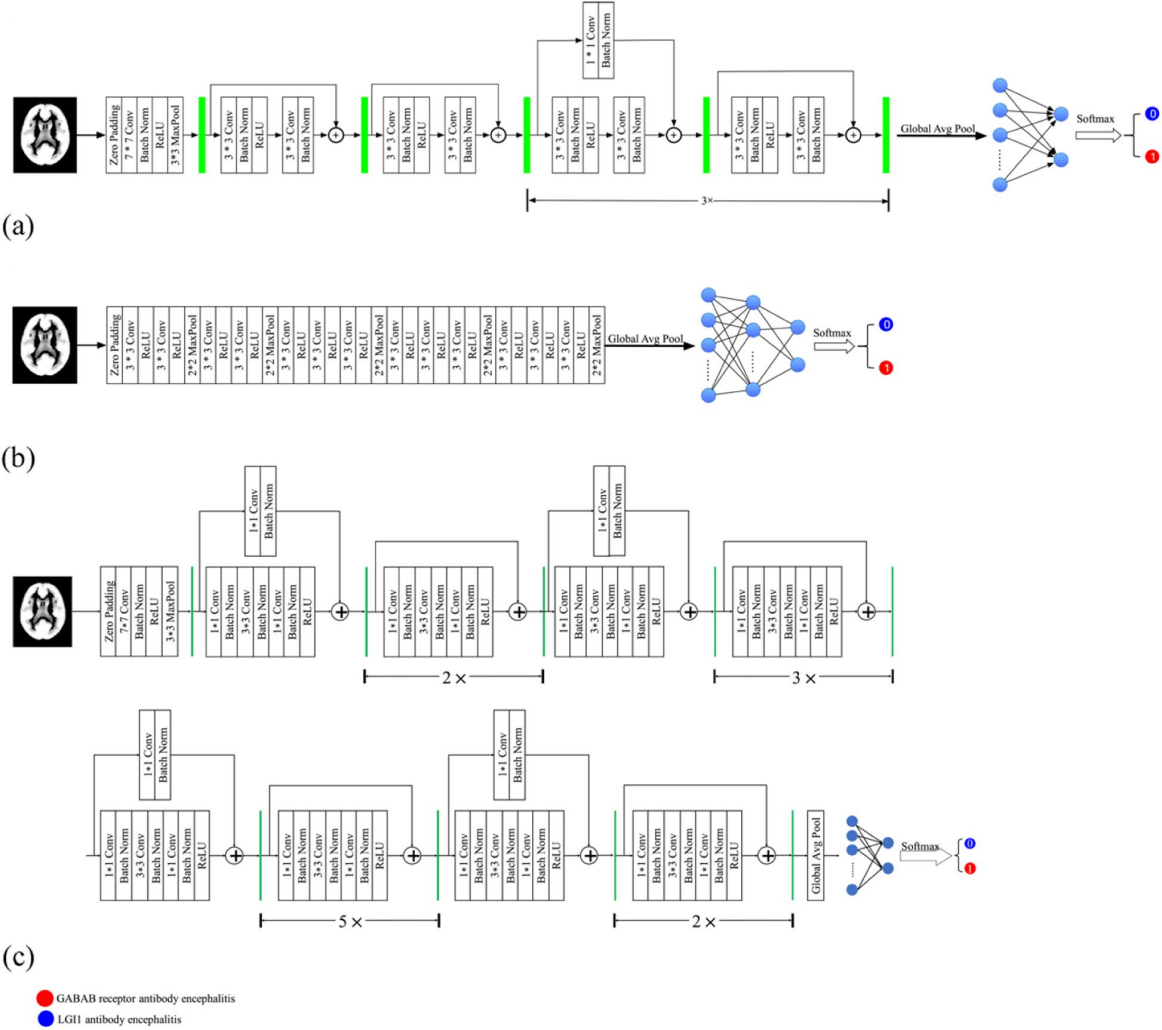
Architecture of the ResNet18 (**a**), VGG16 (**b**) and ResNet50 (**c**). For each of the CNN models, a zero-padding layer was added before the first convolution layer, so that the size of the image fed into the first convolution layer was 224 × 224. The number of the input channel in the first convolution layer was set to one. The output dimension of the fully connected layer was set to two. Conv: Convolution layer; ReLU: Rectified linear unit

In this study, 3828 image slices were used to construct the ResNet18 [30], VGG16 [31], and ResNet50 [30] models. The architectures of the ResNet18, VGG16, and ResNet50 models are shown (Fig. 3). For each CNN model, the number of input channels in the first convolution layer was modified because the image slice was a gray image with only one channel. To match these CNN models in PyTorch (https://pytorch.org/vision/stable/models.html), a zero-padding layer was added before the first convolution layer of each CNN model, so that the size of the image fed into the first convolution layer was 224 $\times$ 224. Because the discrimination between LGI1 and GABAB receptor antibody encephalitis was a binary classification task, the output dimensions of the fully connected layer were set to two.

**Fig. 4:**
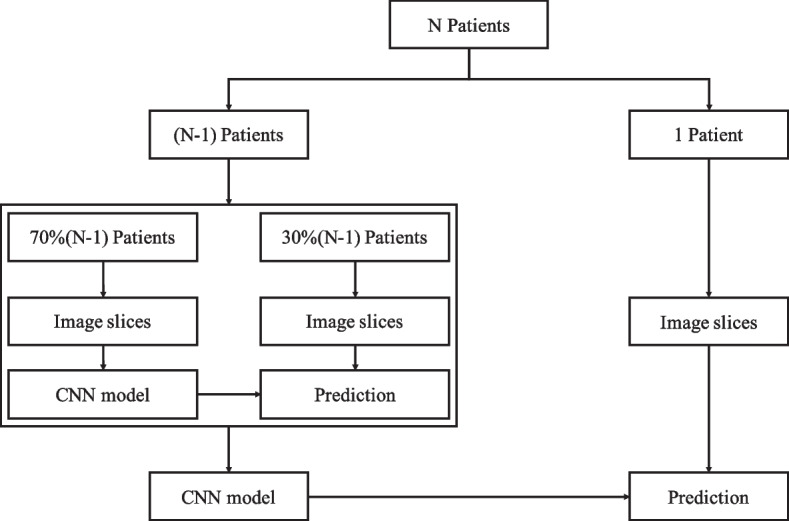
Training and testing of one-fold of LOOCV for the ResNet18, VGG16 and ResNet50 models. In each LOOCV fold, the PET image slices of a patient were used as testing samples (right) and those of the other 80 patients were used as training samples (left)

Given that the number of patients was relatively small, each CNN model was evaluated using leave-one-out cross-validation (LOOCV) at the patient level. As Fig. 4 shows, in each fold of LOOCV, the PET image slices of one patient were used as testing samples (Fig. 4, right), and those of the other 80 patients were used as training samples (Fig. 4, left). This study included 81 patients. Thus, this LOOCV fold was repeated 81 times, with each patient in turn used as the testing cohort and the other 80 patients used as the training cohort. In each LOOCV fold, to reduce overfitting and select the best model, the patients in the training cohort were divided into a training dataset (*n* = 56) and a validation dataset (*n* = 24) in a ratio of 7:3. The image slices of 56 patients in the training dataset were used to train the CNN model, whereas those of 24 patients in the validation dataset were used to select the CNN model with the best performance. Finally, the image slices from one patient in the testing cohort were used to test the selected CNN model.

The ResNet18, VGG16, and ResNet50 models were trained and tested using PyTorch on a Windows computer system. The batch size and the number of epochs were set to 16 and 100, respectively. For ResNet18, the initial learning rate was set to 0.01. The learning rate was updated every five epochs. The new learning rate was calculated by dividing the old learning rate by 10. For VGG16 and ResNet50, the initial learning rate was set as 0.001. The learning rate was updated every 10 epochs. The new learning rate was calculated by dividing the old learning rate by 10. A stochastic gradient descent was used to optimize the model with a momentum of 0.9.

### Performance evaluation at slice level

The number of image slices with GABAB receptor antibody encephalitis detected as GABAB receptor antibody encephalitis was referred to as true positive (TP). The number of image slices with LGI1 antibody encephalitis detected as GABAB receptor antibody encephalitis was referred to as false positive (FP). The number of image slices with LGI1 antibody encephalitis detected as LGI1 antibody encephalitis was referred to as true negative (TN). The number of image slices with GABAB receptor antibody encephalitis detected as LGI1 antibody encephalitis was referred to as false negative (FN).

A total of 81 patients were included. Therefore, the LOOCV consisted of 81 folds. The ${TP}_{i}$, ${FP}_{i}$, ${TN}_{i}$ and ${FN}_{i}$ were the TP, FP, TN and FN of the *i*-th fold of the LOOCV, respectively. *i* (*i* = 1, 2, 3 … 81) represented the *i*-th fold of LOOCV.

Therefore, accuracy was calculated by Eq. 1

1 $$Accuracy= \frac{\sum_{i=1}^{81}({TN}_{i} + {TP}_{i})}{\sum_{i=1}^{81}({TN}_{i} + {TP}_{i}+ {FN}_{i} + {FP}_{i})}\times 100\%$$

sensitivity was calculated by Eq. 2

2 $$Sensitivity = \frac{\sum_{i=1}^{81}{TP}_{i}}{\sum_{i=1}^{81}({TP}_{i}+ {FN}_{i})}\times 100\%$$

specificity was calculated by Eq. 3

3 $$Specificity= \frac{\sum_{i=1}^{81}{TN}_{i}}{\sum_{i=1}^{81}({TN}_{i} + {FP}_{i})}\times 100\%$$

In addition, the receiver operating characteristic (ROC) curve was plotted with 1-specificity and sensitivity on the *x*- and *y*-axis, respectively. The area under the ROC curve (AUC) was calculated to assess the performance of the CNN models at the slice level. In this study, the Delong test was used to compare the ROC curves at the slice level between ResNet18 and VGG16, as well as between ResNet18 and ResNet50.

### Performance evaluation at patient level

**Fig. 5:**
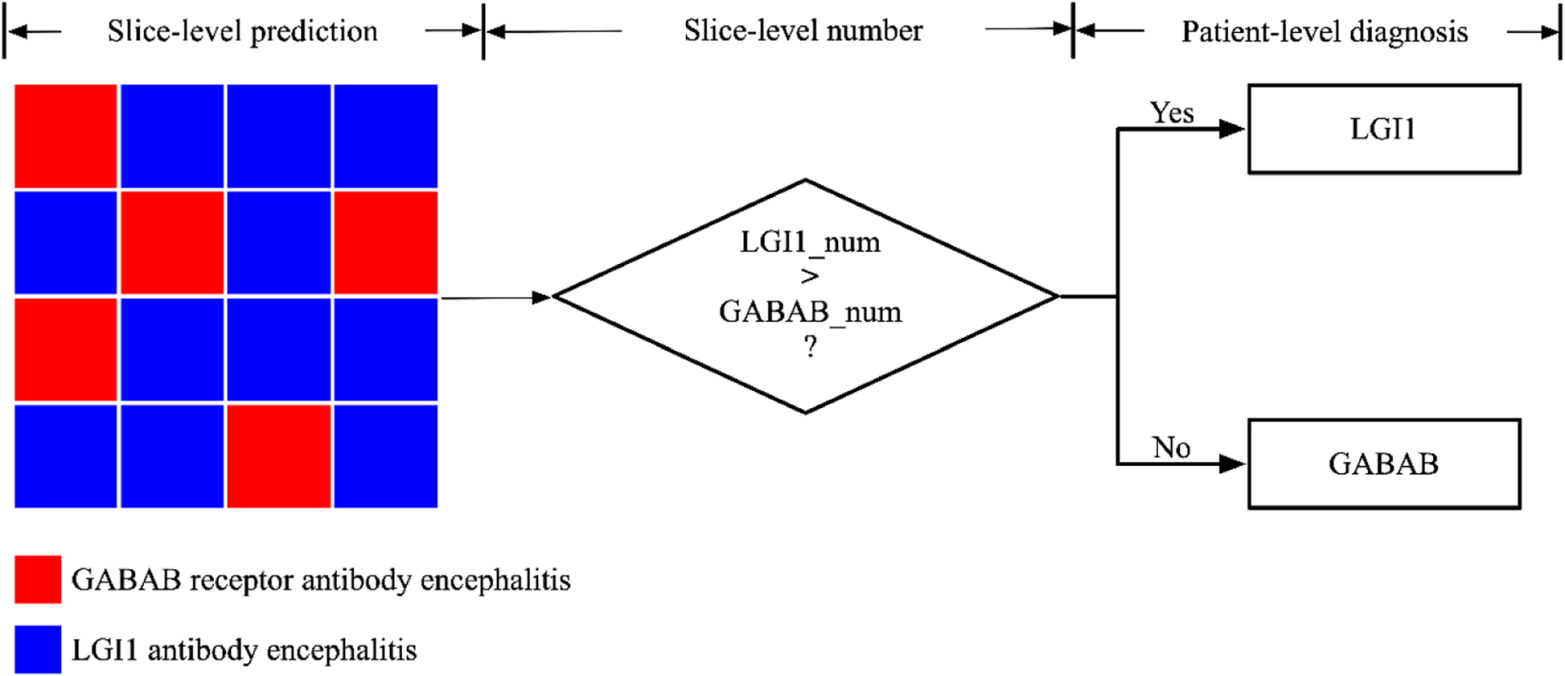
Patient level diagnosis strategy. For a patient, if the number of image slices detected as LGI1 antibody encephalitis (i.e., LGI1_num) was more than that of image slices detected as GABAB receptor antibody encephalitis (i.e., GABAB_num), the patient was detected as LGI1 antibody encephalitis, otherwise detected as GABAB receptor antibody encephalitis. A red rectangle indicated that an image slice was detected as GABAB receptor antibody encephalitis. A green rectangle indicated that an image slice was detected as LGI1 antibody encephalitis

As Fig. 5 shows, a patient-level diagnosis strategy was employed to evaluate the model performance at the patient level. In each LOOCV fold, the image slices of a patient were used as testing samples. If the number of image slices classified as LGI1 antibody encephalitis was larger than that of image slices classified as GABAB receptor antibody encephalitis, the patient was classified as LGI1 antibody encephalitis; otherwise, it was classified as GABAB receptor antibody encephalitis (Fig. 5). After all the LOOCV loops, the prediction results were generated for all patients. Thus, the accuracy was calculated by dividing the number of correct patient predictions by the total number of patients. Sensitivity was calculated by dividing the number of correct predictions in patients with GABAB receptor antibody encephalitis by the total number of patients with GABAB receptor antibody encephalitis. Specificity was calculated by dividing the number of correct predictions in patients with LGI1 antibody encephalitis by the total number of patients with LGI1 antibody encephalitis. In addition, the ROC curve of the patient level was plotted with 1-specificity and sensitivity of the patient level on the *x*- and *y*-axis, respectively. The AUC at the patient level was generated to assess the performance of the CNN model at patient level. In this study, the Delong test was used to compare the ROC curves at the patient level between ResNet18 and VGG16, as well as between ResNet18 and ResNet50.

### Visual explanation of model using gradient-weighted class activation mapping

The best CNN model was selected based on the AUC. Gradient-weighted class activation mapping (Grad-CAM) was used to identify the regions that played important roles in the best CNN model [32]. The gradient of the output of the last fully connected layer with respect to the output (i.e., feature maps) of the final convolution layer was first calculated. Then, the feature maps were multiplied by the average gradient of the corresponding channels. Finally, the summation of all channels of the feature maps was activated using a rectified linear unit function to obtain a heatmap. Because LOOCV was applied, the final heatmap of each image slice was generated from the average of the heatmaps of the image slices across all loops. In this study, the output of the last convolution layer of the best CNN model was employed as the final feature map.

## Results

### Patient demographics

Table 1 summarizes the patient demographics. In total, 81 patients were enrolled in this study. Among these 81 patients, 64 patients with LGI1 antibody encephalitis (58.27 ± 12.62 years, 44 males) and 17 patients with GABAB receptor antibody encephalitis (52.18 ± 12.19 years, 12 males) were verified using antibody testing. However, the weight and height of one patient with LGI1 antibody encephalitis were unknown. Thus, the weight and height of the remaining 63 patients with LGI1 antibody encephalitis were used for statistical analysis. None of the age, gender, weight, and height of patients with LGI1 antibody encephalitis was significantly different from that of patients with GABAB receptor antibody encephalitis with the *p* values of 0.08, 0.88, 0.91, and 0.80, respectively.

### Model performance at slice level and patient level

**Fig. 6:**
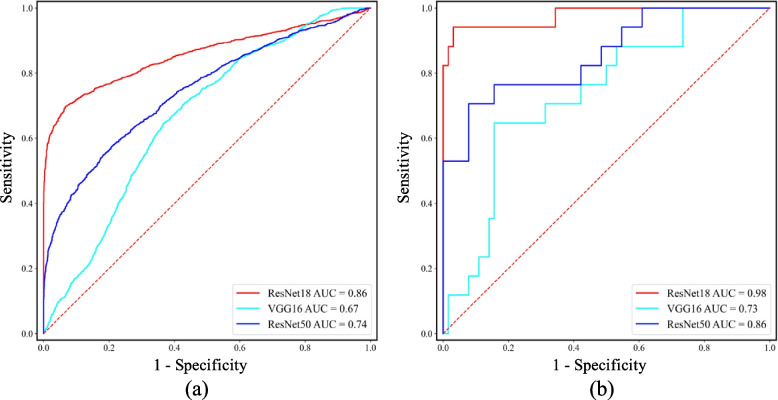
The performance of ResNet18, VGG16 and ResNet50 at slice level (**a**) and patient level (**b**). For the slice level, the sensitivity and specificity refer to the ratio of successfully identifying the image slices with GABAB receptor antibody encephalitis and that successfully identifying the image slices with LGI1 antibody encephalitis, respectively. For the patient level, the sensitivity and specificity refer to the ratio of successfully identifying the patients with GABAB receptor antibody encephalitis and that successfully identifying the patients with LGI1 antibody encephalitis, respectively

Table 2 summarizes the performance of ResNet18, VGG16, and ResNet50 for the classification between image slices with LGI1 antibody encephalitis and those with GABAB receptor antibody encephalitis. Figure 6(a) shows the ROC curves of these models at the slice level. As Table 2 indicates, ResNet18 outperformed VGG16 and ResNet50 at the slice level (*p* < 0.0001). Specifically, the AUC, accuracy, sensitivity, and specificity were 0.86, 80.28%, 73.28%, and 87.72% for ResNet18; 0.67, 62.77%, 77.43%, and 47.20% for VGG16; and 0.74, 67.40%, 52.23%, and 83.51% for ResNet50.

**Table 2:** The classification results of ResNet18, VGG16 and ResNet50 at slice level

	Delong test	AUC	Accuracy	Sensitivity	Specificity
ResNet18	Reference	0.86	80.28% (3073/3828)	73.28% (1445/1972)	87.72% (1628/1856)
VGG16	< 0.0001	0.67	62.77% (2403/3828)	77.43% (1527/1972)	47.20% (876/1856)
ResNet50	< 0.0001	0.74	67.40% (2580/3828)	52.23% (1030/1972)	83.51% (1550/1856)

For the slice level, the sensitivity and specificity refer to the ratio of successfully identifying the image slices with GABAB receptor antibody encephalitis and that successfully identifying the image slices with LGI1 antibody encephalitis, respectively

To evaluate the performance of these network models at the patient level, a decision strategy was employed (Fig. 5), which successfully distinguished between patients with GABAB receptor antibody encephalitis and those with LGI1 antibody encephalitis. Table 3 summarizes the performance of ResNet18, VGG16, and ResNet50 for the classification between patients with LGI1 antibody encephalitis and those with GABAB receptor antibody encephalitis, and Fig. 6 (b) shows the ROC curves of these models at the patient level. As Table 3 indicates, ResNet18 outperformed VGG16 and ResNet50 at the patient level (*p* < 0.01). Specifically, the AUC, accuracy, sensitivity, and specificity were 0.98, 96.30%, 94.12%, and 96.88% for ResNet18; 0.73, 55.56%, 88.24%, and 46.88% for VGG16; and 0.86, 86.42%, 52.94%, and 95.31% for ResNet50, respectively.

**Table 3:** The classification results of ResNet18, VGG16 and ResNet50 at patient level

	Delong Test	AUC	Accuracy	Sensitivity	Specificity
ResNet18	Reference	0.98	96.30% (78/81)	94.12% (16/17)	96.88% (62/64)
VGG16	0.0003	0.73	55.56% (45/81)	88.24% (15/17)	46.88% (30/64)
ResNet50	0.0097	0.86	86.42% (70/81)	52.94% (9/17)	95.31% (61/64)

For the patient level, the sensitivity and specificity refer to the ratio of successfully identifying the patients with GABAB receptor antibody encephalitis and that successfully identifying the patients with LGI1 antibody encephalitis, respectively

### The visual explanation for the model using Grad-CAM

**Fig. 7:**
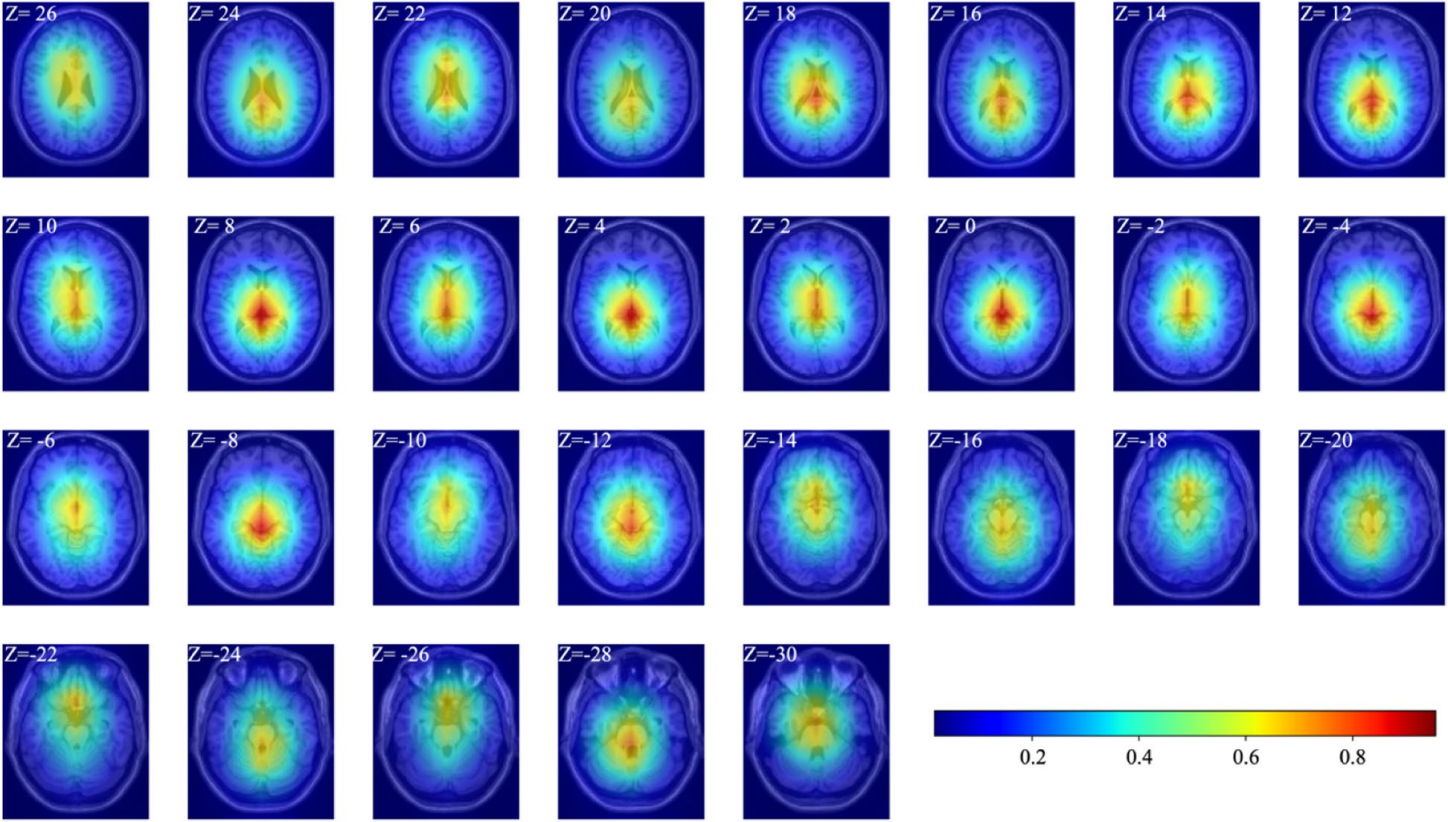
Visual explanation of model using Grad-CAM. The final heatmap of each image slice was generated from the average of the heatmaps of the image slices across all loops. The hot color overlaps on the MTL and BG. The intensity of the hot color indicates the degree with which the model focused on the corresponding region for the discrimination between LGI1 and GABAB receptor antibody encephalitis. The *Z* values in the image slices indicated the MNI coordinates of axial slices

Based on the AUC values, ResNet18 outperformed VGG16 and ResNet50 at the slice and patient levels. In this study, the ResNet18 model was interpreted using Grad-CAM. In Fig. 7, the hot color of a region indicated that the model focused on this region to discriminate between LGI1 and GABAB receptor antibody encephalitis. As Fig. 7 shows, the hot regions overlapped in the majority of the MTL and BG regions, suggesting that the metabolic differences reflected by these two regions might play a more important role in discriminating between LGI1 and GABAB receptor antibody encephalitis.

## Discussion

In this study, ResNet18, VGG16, and ResNet50 models were used to discriminate between LGI1 and GABAB receptor antibody encephalitis. The ResNet18 model achieved the best performance at both slice and patient levels. This study demonstrated that the ResNet18 model could be a prospective approach for discriminating between LGI1 and GABAB receptor antibody encephalitis.

The convolution layer of the CNN models extracted complex and abstract features using a convolution operation between the convolution kernel and the input image. As the number of convolution layers increased, the extracted metabolic features were continuously enhanced and abstracted, which was helpful for identifying subtle changes in medical images. However, as the number of convolution layers in the CNN model increased, the number of parameters increased, requiring more samples to train the corresponding CNN model. Although the number of samples was increased at the slice level, it might not be sufficient to train the ResNet50 model with more parameters than ResNet18. This might be one of the potential explanations for the inferior performance of ResNet50 compared with that of ResNet18. In addition, different from the VGG16, the ResNet18 model employed a “skip connection” (i.e., residual block) to speed up the model training process and improve the accuracy of the classification. A “skip connection” could add features extracted from the previous residual block to the next residual block, preserving as many effective features as possible to discriminate between LGI1 and GABAB receptor antibody encephalitis. Therefore, compared to VGG16, the ResNet18 model achieved better classification performance.

In this study, one of the advantages of using image slices was that the number of samples used to train the models increased. In addition, the 2D ResNet18 model had fewer parameters than the 3D ResNet18 model. Therefore, given the relatively small number of patients, the 2D ResNet18 model with 2D image slices as the input images were trained more easily than the 3D ResNet18 model with 3D images. Therefore, this study employed axial image slices to construct a 2D ResNet18 model to discriminate between LGI1 and GABAB receptor antibody encephalitis. The present study showed that the method using axial image slices achieved a good performance in the classification between patients with LGI1 antibody encephalitis and those with GABAB receptor antibody encephalitis.

Based on the classification results of the ResNet18 model, it was found that the heatmap generated by Grad-CAM included the MTL and BG, indicating that this model focused on the MTL and BG to discriminate between LGI1 and GABAB receptor antibody encephalitis. Multiple studies have revealed that the MTL and BG are common abnormal metabolism regions for LGI1 [13–15] and GABAB receptor antibody encephalitis [16–18], consistent with the findings of this study. Previous studies have attempted to explore the characteristics of PET images to aid in differentiating ALE subtypes. Wegner et al. [33] explored the different metabolic patterns of LGI1 antibody encephalitis from anti-NMDAR encephalitis and found that the BG was hypermetabolic in LGI1 antibody encephalitis. Vedeler and Storstein [34] reported that ALE predominantly affected the MTL, in which hypermetabolism could be detected using FDG-PET. In addition, another study applied a semi-quantitative analysis method for the MTL and BG to increase the detection sensitivity for patients with AE, including patients with LGI1 antibody encephalitis and those with GABAB receptor antibody encephalitis [20]. In this study [20], compared with healthy participants, the metabolism of both the MTL and BG was abnormal in patients with LGI1 antibody encephalitis and those with GABAB receptor antibody encephalitis. This evidence implied that the metabolic pattern was similar for LGI1 and GABAB receptor antibody encephalitis. Thus, it was difficult to discriminate between LGI1 and GABAB receptor antibody encephalitis by the visual assessment of PET images. However, the ResNet18 model could identify subtle differences in the MTL and BG in the PET images.

Notably, the heatmap generated by Grad-CAM did not perfectly focus on the MTL and BG. ResNet18 could learn effective information using a convolution operation between the convolution kernel and image slices. Thus, the method in this study used the global effective information of each image slice to make a decision, highlighting the strength of DL and implying that the method of ‘looking’ medical images was different between DL algorithms and clinicians.

The relatively small number of samples was the main limitation of this study because the incidence of AE was relatively low [35, 36]. Further studies should include more patients with LGI1 antibody encephalitis and those with GABAB receptor antibody encephalitis, particularly prospective cases, to enhance the performance of the DL model. In addition, further studies should focus on identifying the other subtypes of AE.

## Conclusions

In conclusion, the ResNet18 model was a potential approach for discriminating between LGI1 antibody encephalitis and GABAB receptor antibody encephalitis. Metabolism in the MTL and BG was important for discriminating between LGI1 and GABAB receptor antibody encephalitis.

## Data Availability

The original imaging and clinical data are not publicly available, because they contain private patient health information. The result data that support the findings of the present study are available on reasonable requests.

## Abbreviations

LGI1: Leucine-rich glioma-inactivated 1
GABAB: Gamma-aminobutyric acid B
MTL: Medial temporal lobe
BG: Basal ganglia
Grad-CAM: Gradient-weighted class activation mapping
AE: Autoimmune encephalitis
ALE: Autoimmune limbic encephalitis
MRI: Magnetic resonance imaging
PET: Positron emission tomography
DL: Deep learning
ML: Machine learning
CNN: Convolutional neural network
CSF: Cerebrospinal fluid
MNI: Montreal Neurological Institute
LOOCV: Leave-one-out cross-validation
TP: True positive
FP: False positive
TN: True negative
FN: False negative
AUC: Area under the curve
ROC: Receiver operating characteristic
Conv: Convolution layer
ReLU: Rectified linear unit
